# Abundance and Diversity of Hydrogenotrophic Microorganisms in the Infant Gut before the Weaning Period Assessed by Denaturing Gradient Gel Electrophoresis and Quantitative PCR

**DOI:** 10.3389/fnut.2017.00029

**Published:** 2017-06-26

**Authors:** Valeria Sagheddu, Vania Patrone, Francesco Miragoli, Lorenzo Morelli

**Affiliations:** ^1^Facoltà di Scienze Agrarie, Alimentari e Ambientali, Istituto di Microbiologia, Università Cattolica del Sacro Cuore, Piacenza, Italy

**Keywords:** hydrogenotrophs, babies, gut microbiota, *Blautia*, quantitative PCR

## Abstract

Delivery mode (natural vs. cesarean) and feeding type (breast vs. formula feeding) are relevant factors for neonatal gut colonization. Biomolecular methods have shown that the ecological structure of infant microbiota is more complex than previously proposed, suggesting a relevant presence of unculturable bacteria. It has also been postulated that among unculturable bacteria, hydrogenotrophic populations might play a key role in infant health. Sulfate-reducing bacteria (SRB), acetogens, and methanogenic archaea use hydrogenotrophic pathways within the human colon. However, to date, few studies have reported detection of hydrogenotrophic microorganisms in newborns, possibly because of limitations on available group-specific, culture-independent quantification procedures. In the present work, we analyzed 16 fecal samples of healthy babies aged 1–6 months by means of quantitative PCR (qPCR) targeting the 16S rRNA or metabolic functional genes and by denaturing gradient gel electrophoresis (DGGE). qPCR data showed quantifiable levels of methanogens, SRB, and acetogens in all samples, indicating that the relative abundances of these microbial groups were not affected by delivery mode (natural vs. caesarian). DGGE revealed a high prevalence of the *Blautia* genus within the acetogenic bacteria despite strong interindividual variability. Our preliminary results suggest that hydrogenotrophic microorganisms, which have been a neglected group to date, should be included in future ecological and metabolic studies evaluating the infant intestinal microbiota.

## Introduction

Hydrogenotrophic microorganisms inhabiting the gut microbiota of humans and non-human animals are involved in a mutualistic relationship. Three major types of H_2_-consuming microorganisms colonize the human colon, namely, methanogenic archaea, sulfate-reducing bacteria (SRB), and acetogens (1). The common limitation in characterizing these low-abundance microbial communities is related to difficulty in their cultivation, but DNA-based techniques allow detection of microorganisms that are not easily cultured.

Microbiota development is strongly influenced by delivery mode (natural vs. cesarean) and feeding mode (breast vs. formula) (2). To date, only limited and fragmented information is available concerning the influence of delivery and feeding mode on the occurrence of hydrogenotrophic microorganisms. Breastfeeding has been positively associated with CH_4_ production but negatively associated with CH_3_SH and H_2_S (3). On the other hand, soy-based formula has been linked to high CH_4_ and H_2_S levels (3). In an early study, methanogens were detected in 10 out of 40 children (aged 3 months to 10 years), but their levels could not be precisely measured because the 16S rRNA gene density was below the assay’s lower detection limit (4). By using quantitative PCR (qPCR), Palmer et al. (5) detected archaeal 16S rRNA gene sequences in 7 out of 14 samples from 1-year-old infants. Using a PCR assay targeting the *mcrA* gene, a functional gene encoding the methyl coenzyme M reductase, Mihajlovski et al. (6) reported the presence of methanogens in a newborn. Archaeal presence also has been identified in feces samples collected from birth to age 2.5 years (7). Within the archaeal group, *Methanobrevibacter smithii* is the dominant species detected in the human gut microbiota (8). Of interest, *M. smithii* was detected in all fecal specimens collected from 16 children up to the second year of life, including one sample gathered from a 2-week-old infant (9). Previous studies found no evidence of a possible correlation between intestinal archaeal colonization in newborns and dietary habits (10), but a potential, direct mother-to-child transmission has been hypothesized (5).

Concerning SRB, Fite et al. (11) reported their occurrence in 10 infants (aged 12 months) by means of qPCR targeting the 16S rRNA gene. SRB were also detected in 40 newborn stool samples collected from the neonatal period until age 24 months (12). Furthermore, the presence of SRB was reported in 15% of 12 children aged 3 months to 10 years, based on results with a real-time PCR protocol targeting the adenosine-5′-phosphosulfate reductase gene (4). More recently, De Palma et al. (13) described increased values for SRB from age 7 days to 4 months in 20 healthy babies with at least one relative who had celiac disease, as assessed by fluorescent *in situ* hybridization. The distribution of SRB has also been studied using primers targeting the *dsrA* gene, which encodes the alpha subunit of the dissimilatory sulfite reductase (DSR). The ubiquity of DSR in all known SRB microorganisms and its highly conserved nucleotide sequence make this gene suitable for evaluating the quantity and diversity of SRB in the human gut (14). In a recent paper, Pham et al. (15) investigated the occurrence of lactate-utilizing bacteria in 40 term infants, aged 2 weeks to 6 months, delivered either by vaginal birth or a C-section. In this study, SRB were enumerated by both plate count and qPCR. SRB were detected in 71% of infants at 2 weeks by means of plate count, and their levels increased with age. SRB were detected in 33, 23, 19, and 23% of babies at 2 weeks, 1 month, 3 months, and 6 months, respectively, when evaluated by qPCR.

Acetogens are obligate anaerobic bacteria that use the acetyl-CoA (Wood–Ljungdahl) pathway to synthesize acetyl-CoA from CO_2_ and H_2_ (16). Cultivation-based studies have estimated that the number of acetogens in adult human feces ranges from 10^2^ to 10^8^ colony-forming units/g (17). This group consists of metabolically versatile and phylogenetically diverse microorganisms. To date, more than 100 acetogenic species representing 22 genera have been isolated, most belonging to the genera *Acetobacterium* and *Clostridium* (18). In addition, some acetogenic microorganisms isolated from human feces are members of the *Clostridium* cluster XIVa, also known as the *C. coccoides* group (19), which contains a large number of butyrate-producing species (20). Moreover, some species belonging to *Ruminococcus* and *Clostridium* have been reclassified within the genus *Blautia* (21). The characterization of the polyphyletic distribution of acetogens and the identification at the species level using 16S rDNA-based molecular approaches is problematic because of their heterogenic composition. Recently, two functional genes—*fhs*, which encodes formyl tetrahydrofolate synthetase (22), and *acsB*, which encodes acetyl-CoA synthase (23)—have been identified as reliable molecular tools for acetogenic diversity studies. To date, acetogenic bacteria in infant fecal specimens have not been investigated.

Archaeal and bacterial diversity can be characterized using the previously described 515F/806R primer set targeting the V4 region of the 16S rRNA gene (24). However, more recent studies (25, 26) indicate that concentrations around 10^4^ copies per microliter of this target are required to obtain reliable amplicon sequencing data for each bacterial population. In infant fecal samples, Archaeal and SRB levels are likely to fall below this cutoff and, consequently, result in no or reduced PCR product, thereby not allowing the comprehensive profiling of gut microbiota.

The aim of the present study was to assess the presence of hydrogenotrophic populations in the infant gut microbiota of 16 babies aged 1–6 months by means of qPCR and denaturing gradient gel electrophoresis (DGGE). In addition, we evaluated whether or not delivery mode influenced the dynamics of microbial colonization.

## Materials and Methods

### Subjects

Sixteen term infants were investigated (age range: 1–6 months; mean: 2.96 months; SD: 1.35 months), and one fecal sample was collected for each subject. Babies were delivered either vaginally (*n* = 8) or by cesarean section (*n* = 8). No antibiotic treatment was provided during the 4 weeks before analysis. Samples were obtained as part of a previous study performed by Coppa et al. (27). Fecal samples were collected in 2009 at the Department of Pediatrics of the General Hospital of Ascoli Piceno and at the Neonatal Intensive Care Unit, Department of Pediatrics, of the University of Turin. The current study was conducted in compliance with the Helsinki Declaration; each mother signed an informed written consent. The Ethics Committee of the “Ospedali Riuniti” University Hospital, Polytechnic University of Marche, Ancona (Italy), reviewed and approved the study.

### DNA Extraction

Stool samples were stored at −80°C until used. The samples were thawed at room temperature, and total DNA was extracted from 50 mg (wet weight) of feces using the FastDNA™ SPIN Kit for Soil (MP Biomedicals, Switzerland) according to the manufacturer’s instructions (28). Genomic DNA was eluted with 100 µl of elution buffer and its quality verified by agarose gel electrophoresis.

To verify the absence of environmental contamination, a negative control reaction was included in the extraction step. Test sample was replaced by DNA-free water, which underwent the same extraction process that was used for stool specimens. DNA concentration was determined using the Qubit HS dsDNA fluorescence assay (Life Technologies, Carlsbad, CA, USA). Purified DNA was stored at −20°C until used.

### Quantitative PCR

The hydrogenotrophic populations were investigated by means of qPCR with previously described primers (22, 23, 29–32), as reported in Table 1. Six different qPCR assays were performed to specifically quantify total archaea and methanogens (16S rRNA and *mcrA* genes, respectively), SRB (*dsrA* and *aps* genes), and acetogens (*acsB* and *fhs* genes). Additional qPCR reactions were carried out to quantify the *Blautia* genus and the *Clostridium* cluster XIVa, which also includes relevant acetogenic *Blautia* spp (33, 34). and total bacteria (35) (Table 1). All qPCR assays were performed in the StepOnePlus™ Real-Time PCR System (Applied Biosystems Japan, Tokyo, Japan) by using the KAPA SYBR^®^ FAST qPCR Kit Master Mix 2X (Biolab Scientifics Instruments SA, Switzerland) or the KAPA Probe^®^ FAST qPCR Kit Master Mix 2X (Biolab Scientifics Instruments SA, Switzerland). Tenfold serial dilutions of genomic DNA isolated from reference strains were included in each experiment to generate the standard curves; all samples including the negative control for DNA extraction and a no-template control for PCR to confirm the absence of the qPCR water contamination were processed under the conditions reported in Table 1.

**Table 1:** Conditions and thermal protocols for the assessment of quantitative PCR.

Group	Gene	Primer set	Primers and probe, final concentration	Thermal protocol	Standard curve	Reference
Archaea	16S rRNA	ARC787F ARC1059R ARC915F (probe)	400 nM (primers), 100 nM (probe)	94°C 10 s, 60°C 20 s, 45 cycles	g DNA *Methanobrevibacter smithii* DSM 861	(29)

Archaea	*mcrA*	qmcrA-F qmcrA-r-d	400 nM	95°C 10 s, 60°C 40 s, 45 cycles	g DNA *M. smithii* DSM 861	(30)

Sulfate-reducing bacteria (SRB)	*dsrA*	DSR 1-F DSR-R	300 nM	95°C 10 s, 60°C 60 s, 35 cycles	g DNA *Desulfovibrio piger* DSM 749	(32)

SRB	*aps*	aps3F aps2R	400 nM	95°C 10 s, 60°C 40 s, 40 cycles	g DNA *D. piger* DSM 749	(31)

Acetogens	*fhs*	FTHFS_f FTHFS_r	700 nM	95°C 10 s, 55°C 20 s, 72°C 40 s, 40 cycles	g DNA *Blautia producta* DSM2950	(22)

Acetogens	*acsB*	ACS_f ACS_r	500 nM	95°C 10 s, 52°C 20 s, 72°C 30 s, 40 cycles	g DNA *B. producta* DSM2950	(23)

*Blautia* genus	16S rRNA	g-Blau-F g-Blau-R	200 nM	95°C 10 s, 60°C 50 s, 35 cycles	g DNA *B. producta* DSM2950	(33)

*Clostridium* XIVa group	16S rRNA	ErecF–ErecR	400 nM	95°C 10 s, 60°C 50 s, 35 cycles	g DNA *Eubacterium rectale* DSM 17629	(34)

Total bacteria	16S rRNA	Uni 331 F—Uni 797 R	400 nM	95°C 10 s, 60°C 40 s, 40 cycles	g DNA *E. coli* DSM 18039	(35)

### PCR–DGGE

Primers for DGGE analysis of the acetogens group are not available, so we used the primer pair targeting the *acsB* gene described by Gagen et al. (23) and modified the ACS_f primer by adding the GC-clamp. The PCR products obtained with these primers were loaded onto 8% (w/v) polyacrylamide gels (37.5/1, acrylamide/bis-acrylamide) with 20–70% linear DNA-denaturing gradients. Electrophoresis was carried out at 100 V and 60°C for 18 h in an INGENYphor 2 × 2 System (INGENYphor, Goes, Netherlands). We also used the primer pairs described by Maukonen et al. (36) to amplify the V6 region of the 16S rRNA gene of the *C. coccoides–Eubacterium rectale* group, a bacterial cluster that also includes some acetogenic microorganisms, among them *Blautia* spp. The PCR products obtained with the CcocF-GC-CcocR primers (36) were analyzed by DGGE following the methodological conditions described by Sagheddu et al. (37). Predominant bands were excised, re-amplified, sequenced (BMR Genomics, Padova, Italy), and then aligned with the GenBank database (http://www.ncbi.nlm.nih.gov/) by BLAST (38) and the blast algorithm and the Ribosomal Database Project by the Sequence Match tool (39). PCR-DGGE profiles were analyzed by Fingerprinting II SW software (Bio-Rad Laboratories, Hercules, CA, USA). Dendrograms were generated based on the Pearson’s correlation coefficient by means of the Unweighted Pair Group Method with Arithmetic Mean algorithm (UPGMA).

### Statistics

Data normality was checked using the Shapiro–Wilk test, and homoscedasticity was assessed using Levene’s test. Since qPCR data were not normally distributed, they were log-transformed prior to statistical analysis. Differences among the two tested groups were analyzed by means of *t*-test for independent groups (R version 3.1.2) (R Core Team, 2014) (40).

## Results

### qPCR Quantification of Hydrogenotrophic Microorganisms

The cohort of 16 infants under investigation included two different experimental groups based on delivery mode. The groups were as follows: samples 1–8, C-section delivered and samples 9–16, vaginally delivered babies (Table 2).

**Table 2:** Quantification of hydrogenotrophic populations, *Blautia* spp., and *Clostridium* XIVa cluster by quantitative PCR.

Microbial populations	Infant group		*P*-value
	C-section delivered (gene copies/g of wet feces)	Vaginal delivered (gene copies/g of wet feces)	
**Total archaea**			
*16S rRNA gene*	4.52 (0.56)	4.56 (0.45)	0.327
**Methanogens**			
*mcrA*	4.84 (0.28)	4.78 (0.47)	0.429
**Sulfate-reducing bacteria**			
*dsrA*	3.43 (0.38)	3.28 (0.32)	0.543
*aps*	3.15 (0.28)	3.56 (0.31)	0.499
**Acetogens**			
*acsB*	8.61 (0.85)	8.72 (0.74)	0.474
*fhs*	9.49 (0.42)	9.52 (0.51)	0.805
***Blautia***			
*16S rRNA gene*	7.75 (0.88)	8.01 (0.75)	0.339
***Clostridium* XIVa**			
*16S rRNA gene*	9.3 (1.56)	9.75 (1.27)	0.466
**Total bacteria**			
*16S rRNA gene*	11 (0.72)	10.99 (0.55)	0.147

*Results are expressed as the log-transformed mean values of the gene copies per gram of wet feces for each group of infants; SDs in brackets. The last column reports the *P* value obtained by *t*-test for independent groups (*P* < 0.05)*.

Quantitative PCR assays were performed to evaluate the numbers of acetogens, *Clostridium* cluster XIVa, *Blautia* spp., total archaea, methanogens, SRB, and total bacteria in fecal samples. For these bacterial groups, qPCR data indicated that fecal levels were very similar across all infants (Table 2). We found a higher abundance of acetogens, as assessed both by *acsB* and *fhs* gene quantification, compared with both methanogenic archaea and SRB. In addition in our samples, the abundances of *Clostridium* cluster XIVa and *Blautia* spp. were much more homogeneous, suggesting that most of the *Clostridium* cluster XIVa members were represented by species within the *Blautia* genus. No statistically significant difference was found between experimental groups for any bacterial group tested.

### PCR-DGGE Analysis of Acetogenic Bacteria in the Infant Gut

Acetogens emerged as the dominant functional bacterial group among hydrogenotrophic microorganisms in the infant gut, so we attempted to assess the composition of the acetogenic bacterial community through PCR-DGGE analysis targeting the *acsB* gene. This analysis produced DNA bands that could not be identified at the species level although they were all assigned to the genus *Blautia* (data not shown).

**Figure 1:**
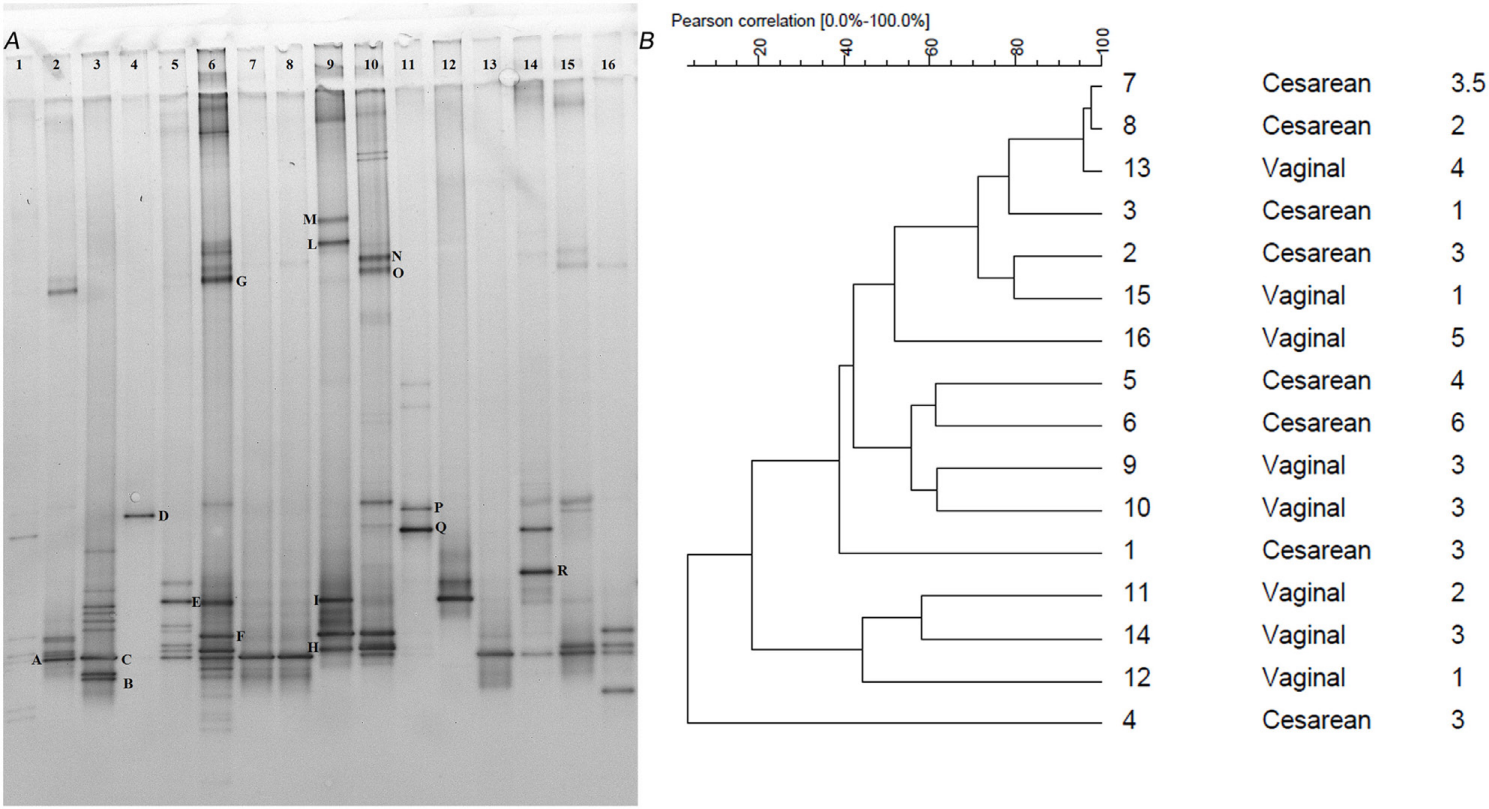
**(A)** PCR-denaturing gradient gel electrophoresis (DGGE) profiles of 16 samples obtained using the primers CcocFGC-CcocR. Samples are subdivided by delivery mode (*n* = 1–8 C-section; *n* = 9–16 vaginal). Bands with letters were sequenced after re-amplification, and the corresponding identities were obtained by alignment in GenBank as reported in Table 3. **(B)** Dendrogram constructed on DGGE patterns by software analysis measured by the Pearson’s correlation coefficient with the UPGMA algorithm. Columns, respectively, indicate delivery mode and infant age expressed in months.

Although the *Clostridium* cluster XVIa is a phylogenetic group that includes many butyrate producers and some acetogenic microorganisms, considering the abundance of the genus *Blautia* in our samples, we attempted to improve the taxonomic classification of the *Blautia* species occurring in our stool samples by using PCR primers targeting the whole *Clostridium* cluster XIVa. The resulting DGGE profiles were markedly different among infants and strongly influenced by interindividual variability rather than by the delivery and feeding conditions (Figure 1). Taxonomic assignment indicated that the genus *Blautia* was prevalent in all fecal samples, with dominant species represented by *Blautia luti, Blautia producta, Blautia wexlerae, Blautia hansenii, Lachnoanaerobaculum orale, Dorea formicigenerans*, and *Ruminococcus gnavus*, as reported in Table 3. Of interest, in one sample, a band corresponding to *Hungatella effluvii* was identified (Table 3). We detected the presence of *B. wexlerae* in eight fecal samples (50%), *B. luti* in two (12.5%), *B. producta* in seven (43.75%), and *B. hansenii* in three (18.75%). The distribution of *Blautia* spp. was not correlated with the delivery mode, thus confirming the qPCR results.

**Table 3:** Identification of bacteria belonging to the *Clostridium* XIVa group based on denaturing gradient gel electrophoresis (DGGE) profiles (see also Figure 1A).

Identification	Bands^a^	Accession number	% Similarity
*Ruminococcus gnavus*	A, C	NR_036800, NR_118690	100
*Hungatella effluvii*	R	NR_133762.1	100
*Lachnoanaerobaculum orale*	D, Q	NR_118086	99
*Blautia luti*	I, L, M, N, O	NR_114315.1	99
*Blautia producta*	F, P	NR_113270	99
*Blautia wexlerae*	H, G	NR_044054	100
*Blautia hansenii*	B	NR_104687.1	99
*Dorea formicigenerans*	E	NR_0044645.2	100

*^a^Bands are lettered as indicated on the DGGE gel shown in Figure 1A*.

## Discussion

Hydrogenotrophic populations are present in the infant intestinal ecosystem, but their detection in infant fecal samples is hampered by their low relative abundance and by experimental limitations related to the recovery of purified high-quality DNA from infant fecal samples. Previous studies conducted on adult humans reported that 30–60% of Western people harbor methanogenic bacteria in their gut (41). More recently, some studies have described higher percentages of adult carriers (42). Methane production during childhood has been demonstrated to start around the third year of life and then to increase with age (43). Stool sample analyses, however, have revealed that methane is produced by 15.3% of 6-month-old infants at concentrations higher than 2 ppm (44). An early study estimated that approximately 10^8^ methanogenic microorganisms per gram of dry weight of stool are needed to generate enough methane to be detectable by breath analysis (45). Additionally, direct competition among methanogens, SRB, and acetogens may occur for the common substrate H_2_. As reported, ~50% of healthy humans carry intestinal SRB, and several studies have described their presence at different ages in the gut lumen (4, 46) and the distal gut mucosa (14). In addition, SRB are postulated to be positively associated with inflammation (47). In contrast, few studies have assessed the presence and distribution of acetogenic bacteria in the human gastrointestinal tract.

A deep understanding of the diversity of bacteria inhabiting the infant gut microbiota is relevant for human gut ecology and future nourishment research. The major result of our study was that all infants harbored detectable levels of methanogens, acetogens, and SRB, as evaluated using specific qPCR for the 16S rRNA gene or the functional metabolic genes *mcrA, acsB, fhs, dsrA*, and *aps*. We detected about 10^4^–10^5^ copies/g of feces of the 16S rRNA (total archaea) and *mcrA* genes (methanogens), respectively, 10^3^–10^4^ copies/g of feces of the *dsrA* and *aps* genes (SRB), and 10^8^–10^9^ copies/g of feces of the *acsB* gene, and 10^9^–10^10^ copies/g of feces of the *fhs* gene (acetogens), respectively. Considering the numbers of total bacteria present in our samples (about 10^10^–10^11^ 16S rRNA gene copies/gram of feces), hydrogenotrophic microorganisms and in particular acetogens seem to represent a not negligible fraction of the infant gut microbiota.

The consistent recovery of hydrogenotrophic populations in all samples probably depended on the DNA extraction method performed or primer pairs used for the qPCR analyses. As Dridi et al. emphasized (9), the DNA extraction method exerts great influence on the recovery of archaea, suggesting that mechanical lysis with beads could overcome the presence of the K-resistant proteinase cell wall of methanogenic archaea. As in our study, Fite et al. (11) and Hopkins et al. (12), using a bead beating extraction protocol, recovered the presence of SRB in all fecal samples of babies tested. With a different DNA extraction system, Stewart et al. (4) reported the presence of methanogens and SRB, respectively, in 25 and 15% of fecal samples using previously described primers targeting the 16S rRNA (48, 49) and the *aps* (50) gene, respectively. With the same DNA extraction protocol, Palmer et al. (5) detected about 10^3^–10^6^ archaeal 16S rRNA gene copies/gram of wet feces in 7 out of 14 fecal samples using universal primers for the 16S rRNA gene (51, 52). These inconsistencies suggest that further efforts are needed to develop reliable molecular tools for hydrogenotrophic microorganism investigations. Notably, a recent review by Carbonero et al. (53) concluded that the occurrence of the functional genes *acsB* and *fhs* is not automatically associated to reductive acetogenesis. On the contrary, *mcrA* and *dsrA* genes seem to be reliable markers of both relative abundance and activity of methanogenic and SRB communities, respectively.

Comparison between the two DGGE analyses revealed that the 16S rRNA gene better described the complexity of the acetogenic distribution compared to the functional gene *acsB*. The DGGE analyses of the 16S rRNA gene allowed for identification of a greater number of acetogenic bacteria species compared to the DGGE for the *acsB* gene. In contrast, it was not possible to perform the DGGE analyses for the SRB and methanogens because their levels fell below the detection limit of PCR-DGGE, which ranges from 10^4^ to 10^8^ colony-forming units/ml (54). As a result, the presence of these microorganisms was quantifiable by qPCR, but conventional PCR did not produce any amplification fragment. In a recent review, Flint et al. (55) pointed out the necessity of investigating functional rather than phylogenetic marker genes. The study of metabolism-related genes will provide additional information allowing a comprehensive analysis of the gut microbiota. Unfortunately, in our case, only the DGGE analysis of the 16S rRNA gene gave positive results.

In this study, we failed to detect *Blautia* spp. 16S rRNA gene sequences by DGGE in only 1 of the 16 stool specimens, and in 2 out of 16, the band corresponding to the *Blautia* genus was faint; nevertheless, the identification of this genus was possible by qPCR. The *Blautia* genus was recognized in 93.75% of infants by DGGE and in all samples by qPCR in babies under age 6 months. Our results are in agreement with those reported by Kurakawa et al. (33), who described *Blautia* spp. predominance in the human intestinal *Clostridium* XVIa group regardless of age in three groups (32 children aged 3.2 ± 0.1 years, 32 healthy adults aged 39 ± 11 years, 32 healthy elderly aged 82 ± 6 years) (33). In recent work, Touyama et al. (56) described the presence of *B. wexlerae* and *B. luti* in the fecal samples of 12 healthy Japanese adults. The present study underlines the real problem of the possible underestimation of neglected hydrogenotrophic populations.

Another interesting finding was the recovery of a band corresponding to *H. effluvii*. This microorganism was recently isolated from an industrial treatment plant (57), and to the best of our knowledge, this study is the first time that it has been recovered in infant human samples. The difficulty of detecting these microorganisms is higher in infant specimens compared to adult samples, mainly because of lower levels of these microorganisms in infant specimens.

This pilot study also presents some limitations that require mention. First is the small number of recruited infants, and further studies on larger cohorts of babies are needed to define the putative role of hydrogenotrophic microorganisms in the infant gut microbiota. Since our study involved only a restricted number of samples, the major inferences that can be drawn from our results are related to the methodological approaches which allowed us to detect hydrogenotrophic populations at a quantifiable level in all infant fecal samples. The possibility of enumerating and identifying hydrogenotrophic species would be a major goal. To achieve such an aim, the punctual setting of DNA extraction protocols and the development of highly specific and efficient sets of primers with a good coverage would represent a crucial step to gain deeper insight into their actual ecological impact.

## Ethics Statement

The current study was conducted in compliance with the Helsinki Declaration; each mother signed an informed written consent. The Ethics Committee of the “Ospedali Riuniti” University Hospital, Polytechnic University of Marche, Ancona (Italy), reviewed and approved the study.

## Author Contributions

VS drafted the manuscript, collected samples, and performed DNA extraction and quantitative PCR. VP jointly led the study and revised the manuscript. FM performed the denaturing gradient gel electrophoresis and the cluster analysis and revised the manuscript. LM conceived and designed the study and revised the manuscript. All the authors read and approved the final manuscript.

## Conflict of Interest Statement

The authors declare that the research was conducted in the absence of any commercial or financial relationships that could be construed as a potential conflict of interest.
